# Management in primary care at the time of a suicide attempt and its impact on care post-suicide attempt: an observational study in the French GP sentinel surveillance system

**DOI:** 10.1186/s12875-020-01126-9

**Published:** 2020-03-25

**Authors:** Nadia Younes, Mathieu Rivière, Frédéric Urbain, Romain Pons, Thomas Hanslik, Louise Rossignol, Christine Chan Chee, Thierry Blanchon

**Affiliations:** 1 EA 40-47 Université Versailles Saint-Quentin-en-Yvelines, F-78047 Guyancourt, France; 2 Versailles Hospital, Academic Unit of Psychiatry, 177 Rue de Versailles, 78157 Le Chesnay, Cedex France; 3 Université Versailles-Saint-Quentin-en-Yvelines, Versailles, France; 4 Sorbonne Université, INSERM, Pierre Louise Institute of Epidemiology and Public Health, F-75013 Paris, France; 5 Infectious Disease Department, CHR Orléans La Source, Orléans, France; 6 French Institute for Public Health Surveillance, Saint Maurice, France

**Keywords:** Suicide prevention, General practice, Suicide attempts, Management of the suicide attempt, Post-suicide attempt care

## Abstract

**Background:**

We aimed to describe primary care management at the time of a suicide attempt (SA) and after the SA.

**Methods:**

An observational (cross-sectional) study was conducted among 166 sentinel GPs within France (a non-gatekeeping country) between 2013 and 2017 for all GP’s patients who attempted suicide. Measurements: frequency of patients 1) managed by the GP at the time of the SA, 2) addressed to an emergency department (ED), 3) without care at the time of the SA, and 4) managed by the GP after the SA and factors associated with GP management at the time of and after the SA.

**Results:**

Three hundred twenty-one SAs were reported, of which *N* = 95 (29.6%) were managed by the GP at the time of the SA, N = (70.5%) were referred to an ED, and N = (27.4%) remained at home. Forty-eight (14.9%) patients did not receive any care at the time of the SA and 178 (55.4%) were managed directly by an ED. GPs were more likely to be involved in management of the patient at the time of the SA if they were younger (39.2% for patients < 34 years old; 22.9% for those 35 to 54 years old, and 30.3% for those more than 55 years old *p* = 0.02) or the SA involved a firearm or self-cutting (51.9%) versus those involving drugs (23.7%); *p* = 0.006). After the SA, GPs managed 174 patients (54.2%), more often (60%) when they provided care at home at the time of the SA, *p* = 0.04; 1.87 [1.07; 3.35]. No other factor was associated with management by GPs after the SA.

**Conclusions:**

The study faced limitations: data were not available for patients managed solely by specialists during their SA and results may not be generalisable to countries with a stronger gatekeeping system. We concluded that GPs are involved in the management of patients at the time of a SA for a third of patients. EDs are the major provider of care at that time. Half patients consulted GPs after the SA and connections between GPs and ED upon discharge should be improved.

## Background

Suicidal behaviour has been identified as a major public health issue worldwide [1]. In France, 195,000 suicide attempts (SA) [2, 3] and 10,000 suicides (incidence rate 18.0/100,000 inhabitants) [4] are reported per year. This is among the highest incidence rates in Europe.

GPs play a central role in suicide prevention and the management of suicidal patients. Suicidal behaviours are frequent in primary care: 2.4 to 8% of primary care patients have suicidal thoughts [5, 6] and a GP encounters one to six SAs in a working year [7, 8] and loses a patient by suicide every four to 7 years [7, 9]. Among patients with depressive disorders, 10.4% attempted suicide during the last 5 years [10]. At least half of patients who died by suicide and two thirds of those who attempted suicide visited a GP in the preceding months, challenging the GPs’ recognition and management of suicidal patients [11–13]. According to Milner’s systematic review and meta-analysis, suicide prevention in primary care produced equivocal results [14]. One point has not yet been investigated: the involvement of primary-care professionals *at the time of the SA*. The role of emergency departments (EDs) has been more explored. A systematic review estimated that mental or behavioural-health disorders accounted for 4% of ED attendance and, among these, a third due to self-harm or suicidal ideation [15]. In standard practice in the ED, those who attempt suicide are either hospitalized or sent home after treatment and evaluation by a mental-health professional, with some variability in the organization according to country and setting [14–17]. We found few data on prehospital or primary care. A South-African study explored the attitudes of 130 prehospital providers (not including GPs) on transport decision in the management of SA patients who refuse care. They reported a critical lack of training and certain negative attitudes and difficulties [18]. For primary care, the position of GPs (patients’ expectations and GPs’ task performance regarding mental health care, role of others health actors) varies between health care systems. In more gatekeeping countries (as in Netherlands, Spain and the United Kingdom for example), a patient cannot consult a medical specialist directly and GPs’ task could be different than in non-gatekeeping countries (as in Belgium, France, Germany, Canada and Switzerland) where patients may have direct access to specialists [19]. The Belgian Network of Sentinel General Practices reported several characteristics of SA encountered between 2013 and 2016 (*n* = 245), including one information about GP informant of suicide attempt (*n* = 241) and established that GP was on site as first caregiver following the SA for *N* = 46 patients (19.1%) [20]. The first aim of our study was to describe further, in another non-gatekeeping country, the role of GPs as first caregiver following the SA (how often, for which patients, and their management: how often GPs refer patients to the hospital, how often they give care in place of the hospital).

The aftercare of a SA is challenging, because of the high risk of subsequent suicide or all-cause mortality [21] and morbidity. Two of 10 patients repeat a SA during the first 5 years [22]. Active contact and follow-up has been shown to be effective in preventing a repeat SA in the first 12 months (*n* = 5319; pooled RR = 0.83; 95% CI: 0.71 to 0.97). However, the effect at 24 months was not confirmed in EDs [23]. Studies conducted among patients registered in a general hospital in the UK for SA have reported the central role of primary care (gatekeeping system): approximately 30 to 40% consulted their GP in the week following a SA and 25 to 50% in the following month. Among them, 58% discussed the SA during the first consultation [7, 11, 24]. The rate of mental health treatment does not exceed 30–50%, with a higher rate if the appointment was booked with a mental health professional before discharge [25]. A recent study conducted in Canada a non-gatekeeping country, reported a more limited role of primary care and a high frequency of patients without any care: among 23,140 individuals attending EDs for self-harm in a Canadian hospital, 10.7% consulted a GP for a mental-health visit within 1 month, 17.1% a psychiatrist, 3.6% both, and 68.6% had no care [22]. The role of primary care needs to be explored further. We aimed to describe how often and for which patients GPs provide aftercare following a SA and whether the role of GPs as first caregiver following the SA is associated with more aftercare by the GP.

The objective of the present study is to describe primary care management 1) at the time of a SA and 2) after the SA.

## Methods

### Population and procedure

We studied “suicidal” patients from the French General Practice Sentinel Network from January 1, 2013 to December 31, 2016. The study was authorized by the scientific board of the Network. During the study period, 601 GPs throughout metropolitan France had continually reported online the occurrence of 10 health related events (including SA) on an unpaid volunteer basis at some point in their work week [26]. Sentinelles GPs practise like other French GPs. They are not different from other in term of age and practice of complementary medicine but slightly different regarding location, gender (female: 19% vs 29%) and number of consultation per week (94 vs 92) [27]. We hypothesized that those minor differences won’t have a major impact on our results regarding generalization to other SA seen by French GPs. For SA, GPs are instructed to report all cases they are confronted with in their daily practice, namely individuals seen while they are on duty or seen by other caregivers (mainly EDs) who belong to the GPs patient base. SAs are defined, from the WHO/EURO para-suicide definition of “suicidal acts of self-inflicted injury or self-poisoning with drugs in excess of the generally recognized therapeutic dose, excluding non-suicidal self-injury or self-poisoning” [28, 29].

The GPs report:

- the patients’ sociodemographic data (for age, we considered three classes: < 34 years, 35 to 54 years, and > 55 years) and clinical characteristics
- characteristics of the last consultation in primary care (time, expression of suicidal ideation) and their previous management: a) the presence of “psychological difficulties” or “depression” in the preceding year and the b) provision of psychological support, psychotropic drugs, referral to a psychiatrist or psychologist, and specialized care during the preceding 3 months,
- whether they managed the patient at home at the time of the SA,
- whether they managed the patient after the SA.

For this study, we considered only SAs (excluding SA that lead to suicide).

### Analysis

**Fig. 1:**
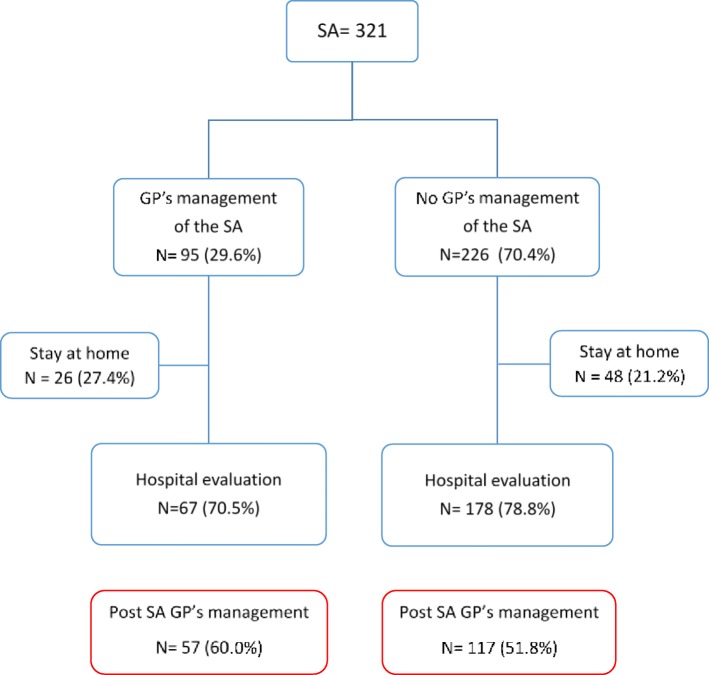
Patients managed by GPs at the time and after the suicide attempt (SA)

Chi-square or Fischer tests were carried out to test for significant differences between GPs reporting SA and GPs not reporting SA in the French General Practice Sentinel Network. Descriptive analyses were used to report the management of the patients by the GP (Fig. 1). Chi-square or Fischer tests were carried out to test for significant differences between SAs managed by the GP or not, according to the patients’ sociodemographic and clinical characteristics. Similarly, tests were carried out for post-SA management by GPs. We also computed odds ratios with a 95% confidence interval. *P*-values < 0.05 were considered to reach statistically significance. All analyses were performed using GNU R software, version 3.1.1 [30].

### Ethics statement

The French Sentinel general practice Network is approved by the National Data Protection Agency (CNIL, registration number #47139). The protocol was conducted in agreement with the Helsinki Declaration. All GPs participants are volunteers to participate and they fulfilled questionnaires in line. They are informed about studies conducted from their medical charts. We performed an observational study on anonymous data without any way to identify patients. Patients are informed that their GP belongs to the Network. The ethics committee Comité de protection des personnes Ile de France V” « surveillance épidémiologique du réseau Sentinelles-C-11-15 » approved this procedure.

## Results

From January 2013 to December 2016, 321 SAs in primary care (for 312 patients estimated) were reported to the Sentinel network by 166 GPs. *N* = 435 GPs did not encounter SAs among their patients at that time.

### GPs’ reporting (Table 1)

In the Sentinel Network, GPs not reporting SA (*N* = 435) and GPs reporting SA (*N* = 166, with *N* = 95 GPs, 57.2% reported one SA, *N* = 35, 21.1% reported two and *N* = 36, 21.7% thee and more; *N* = 156 GPs had a 2 months follow-up after the SA) did not differ. GPs were mainly men, about 50 y.o., with urban and group practice. They came from all the 13 administrative area in France.

**Table 1:** Comparison of GPs reporting SA (*N* = 166) and GPs not reporting SA (*N* = 435) in the French General Practice Sentinel Network in 2013–2016 (*N* = 601)

	GPs reporting SA ***N*** = 166 27.6% N (%)	GPs not reporting SA ***N*** = 435 72.4% N (%)	***p***
Sex			ns
Female	49 (29.5)	117 (26.9)	
Male	117 (70.5)	318 (73.1)	
Age in 2013 median (q1-q3)	52 (41–58)	53 (42–59)	ns
Length of medical practice (in 2013) median (q1-q3)	19 (5–17)	21 (7–29)	ns
Length of Sentinel practice (in 2013) median (q1-q3)	5 (1–15)	6 (0–15)	ns
Medical practice			ns
Solo practitioner	53 (36.6)	131 (40.3)	
Group practitioner	92 (63.4)	194 (59.7)	
Practice setting			ns
Urban	126 (75.9)	346 (79.7)	
Rural	40 (24.1)	88 (20.3)	
Region in France			ns
Auvergne Rhone Alpes	42 (25.3)	93 (21.4)	
Bourgogne Franche Comté	13 (7.8)	13 (3.0)	
Bretagne	12 (7.2)	21 (4.8)	
Centre Val de Loire	13 (7.8)	89 (48.1)	
Corse	27 (38.0)	24 (5.5)	
Grand Est	11 (6.6)	39 (9.0)	
Hauts de France	11 (6.6)	28 (6.4)	
Ile de France	16 (9.6)	58 (1.3)	
Normandie	6 (3.6)	18 (4.1)	
Nouvelle Aquitaine	8 (4.8)	29 (6.7)	
Occitanie	22 (13.2)	39 (9.0)	
Pays de Loire	4 (2.4)	17 (3.9)	
Provence-Alpes-Côte d’Azur	5 (3.0)	31 (7.1)	

### Patients managed by GPs at the time of the SA and after (Fig. 1)

GPs were involved in the management of 95 (29.6%) patients at the time of their SA. They referred most to the hospital for evaluation and management, but 27.4% stayed home. GPs also reported that 14.9% of their patients who attempted suicide (*N* = 48) stayed home without care following their SA and 55.4% (*N* = 178) were managed at the hospital without their intervention.

Globally, hospital management was central for 245 (76.3%) patients in primary care at the time of the SA (patients addressed to the ED by GPs or patients presenting directly to the ED). GPs reported a connection (written or oral) with the hospital for *N* = 115 patients (46.9%).

After the SA, GPs managed the care of 174 patients (54.2%), more often (60%) if they provided care at home at the time of the SA, *p* = 0.04; 1.87 [1.07; 3.35].

### Factors associated with care by GPs at the time of the SA and after (Tables 2 and 3)

At the time of the SA, GPs were more often involved in the care of their patients if they were younger (39.2% for patients aged between 18 and 34 years vs 22.9% for those aged 35–54 years and 30.3% for those older than 55 years; *p* = 0.02, with respective ORs of 1, 0.47 [0.26–0.81], and 0.68 [0.35–1.28]) and if the method of the SA was self-cutting versus drugs (OR = 3.44 [1.50–7.94]; *p* = 0.06). No other factors (sex and clinical characteristics, expression of suicidal ideas at the last consultation, or previous mental health management in primary care) were associated with the patient being managed by a GP at the time of the SA.

**Table 2:** Comparison of patients who attempted suicide managed or not by GPs at the time of the suicide attempt (*N* = 321)

	Managed by a GP at the time of the SA *N* = 95 29.6% N (%)	Not managed by a GP at the time of the SA *N* = 226 70.4% N (%)	*p*	OR [95% CI]
Sex (*n* = 318)			ns	
Female	47 (50.0)	127 (56.7)		1
Male	47 (50.0)	97 (43.3)		1.31 [0.81–2.13]
Age(*n* = 321)			**0.02**	
< 34 yo	38 (40.0)	59 (26.1)		1
35–54 yo	34 (35.8)	114 (50.4)		0.47 [0.26–0.81]
≥ 55 yo	23 (24.2)	53 (23.5)		0.68 [0.35–1.28]
Suicidal methods used (*n* = 308)			**0.006**	
Drugs	51 (56.7)	164 (75.2)		1
Self-cutting	14 (15.5)	13 (6.0)		3.44 [1.50–7.94]
Hanging and firearm	8 (8.9)	15 (6.9)		1.72 [0.65–4.25]
Others (downing, falls, etc)	17 (18.9)	26 (11.9)		2.10 [1.04–4.17]
History of previous attempts (*n* = 286)	36 (42.4)	82 (40.8)	ns	1.07 [0.63–1.78]
Suicidal ideas expressed at the last consultation (*n* = 260)	34 (46.6)	79 (42.2)	ns	1.19 [0.69–2.06]
Time of the last consultation (*n* = 261)			ns	
< 1 month	45 (60.8)	102 (54.5)		1
≥ 1 month	29 (39.2)	85 (45.5)		0.77 [0.44–1.34]
GP management in the last three months (*n* = 262)				
Psychological support	32 (44.4)	80 (43.4)	ns	1.02 [0.59–1.77]
Antidepressant prescriptions	26 (36.1)	75 (41.0)	ns	0.82 [0.46–1.43]
Other psychotropic drug prescriptions	32 (44.4)	99 (53.2)	ns	0.70 [0.40–1.22]
Attempted referral to a mental-health specialist	33 (47.1)	89 (48.1)	ns	0.96 [0.55–1.67]
Parallel care with a mental health specialist	27 (38.0)	73 (39.2)	ns	0.95 [0.54–1.67]

**Table 3:** Comparison of patients who attempted suicide managed or not by a GP after the suicide attempt (SA) (*N* = 281)

	Managed by a GP after the SA *N* = 174 61.9% N (%)	Not managed by a GP after the SA *N* = 107 38.1% N (%)	*p*	OR [95% CI]
Sex (*n* = 279)			ns	
Female	94 (54.0)	62 (59.0)		1
Male	80 (46.0)	43 (41.0)		1.22 [0.75–2.01]
Age (*n* = 281)			ns	
< 34 yo	50 (28.7)	34 (31.8)		1
35–54 yo	79 (45.4)	57 (53.3)		0.94 [0.54–1.64]
> = 55 yo	45 (25.9)	16 (14.9)		1.90 [0.93–3.98]
Suicidal methods used (*n* = 270)			ns	
Drugs	111 (66.9)	79 (76.7)		1
Self-cutting	16 (9.6)	8 (7.8)		1.41 [0.58–3.67]
Hanging and firearm	15 (9.0)	4 (3.9)		2.59 [0.89–9.62]
Others	24 (14.5)	12 (11.6)		1.41 [0.68–3.10]
History of previous attempts (*n* = 251)	62 (38.7)	41 (45.0)	ns	0.77 [0.46–1.30]
Suicidal ideas expressed at the last consultation (*n* = 228)	69 (48.9)	34 (39.1)	ns	1.49 [0.87–2.58]
Time of the last consultation (*n* = 228)			ns	
< 1 month	84 (59.6)	43 (49.4)		1
≥ 1 month	57 (40.4)	44 (50.6)		0.66 [0.39–1.14]
GP management in the last three months (*n* = 230)				
Psychological support	66 (47.8)	32 (38.1)	ns	1.48 [0.85–2.60]
Antidepressant prescriptions	59 (41.8)	34 (40.0)	ns	1.10 [0.64–1.93]
Other psychotropic drug prescriptions	76 (54.3)	38 (43.7)	ns	1.53 [0.89–2.63]
Attempted referral to a mental-health specialist	66 (48.2)	43 (50.0)	ns	0.93 [0.54–1.60]
Parallel care with a mental-health specialist	59 (42.4)	31 (35.6)	ns	1.33 [0.77–2.33]
Managed by a GP at the time of the SA (*n* = 281)			0.04	
No	117 (67.2)	85 (79.4)		1
Yes	57 (32.8)	22 (20.6)		1.87 [1.07–3.35]

No factor was associated with the SA patients staying at home without care or those addressed by GPs to hospitals.

Post-SA patients managed by a GP were not associated with factors, except when the SA was managed by the GP at the time of the SA. Patients managed by the GP at the time of the SA were more likely to have aftercare by the GP, *p* = 0.04; 1.87 [1.07; 3.35].

## Discussion

GPs were first caregiver following the SA for 29.6% of their patients (more if the patients were younger or they used self-cutting for their SA). They referred two-thirds to hospitals and one third remained at home. Connections between GPs and ED upon discharge occurred for 46.9% of patients.

After the SA, GPs were consulted by 54.2% of their SA patients, more often when GPs were first caregiver following the SA.

### GP as first caregiver following the SA

We found a similar (event slightly higher) percentage than in the study conducted in another non-gatekeeping country by the Belgian Network of Sentinel General Practices [20]. It concerns a minority of patients: most SAs were managed by other prehospital providers, such as firemen or family [18] and EDs [15]. It is interesting to note that most patients managed by their GP at the time of SA were referred to the hospital. Our study was quantitative and did not explore difficulties encountered by GPs as prehospital or first providers. GPs may face certain difficult situations, such as patients refusing care or having limited confidence to make decisions in such situations, as described elsewhere among other providers [18].

GPs were more frequently the first caregiver for younger patients and in cases of SA using self-cutting, compared to self-poisoning which was more associated with hospital management and admission [31].

We did not find any factors that differentiated between patients who stayed at home and those addressed to hospitals for SAs managed by the GPs at home, possibly due to a lack of statistical power.

Our study also measured the frequency of patients who attempted suicide who did not seek help (approximately 15%), which has not yet been explored in the context of primary-care recruitment. A higher rate (26.8%) has been reported for the general French population by the Baromètre Santé [32]; the difference lies in the survey methodology and target population. Moreover, GPs in our survey may have been unaware of some SAs. One of the major obstacles hindering the help-seeking process is the refusal by the suicide attempter to receive professional care and the “help-seeking road travelled by the significant others of a suicide attempter is, in most cases, tortuous and difficult” [33]. Patients may conceal suicidality because of stigmatization [34]. More women seek help [32] but we did not find this result in our survey.

### Patient management by GPs after the SA

Our results confirmed the central place of primary care in the aftercare of SA patients [7, 11, 24], but also the high frequency of those who reveive no care after a SA [22]. Brief contact interventions for any patient leaving the ED after a SA, to help to cope with any new suicidal crisis could be reliable suicide prevention strategies, could be reliable suicide prevention strategies, in collaboration with GPs [23, 35, 36].

We did not find any factors associated with such aftercare, except whether the patient was managed by the GP at the time of the SA, with a higher frequency of patients who received aftercare by the GP if the GP was the first caregiver following the SA. This result may be related to a stronger physician-patient-family working alliance [37, 38].

Connection between GPs and ED upon discharge (47%) is lower than found in another non-gatekeeping country [20] and should be improved as GPs are central after the SA.

### Strengths and limitations of the study

The main strength of this study was the data from the Sentinelles Network, which allowed the assessment of all SAs encountered in the primary-care setting over a four-year period.

Our study also had several limitations. First, a selection bias. SAs may have been underreported by GPs, who forget to report them or may not have been aware of some suicidal acts (for suicide attempts that did not lead to a medical intervention, especially among patients less involved in primary care and with less aftercare [7, 9, 10, 39, 40]). Participation was, however, comparable to that reported for the two other existing General-Practice Sentinel Networks [9, 20, 41, 42]. Second, Sentinelles GPs may not reflect the practices of French GPs, as their involvement in the Network may make them more aware of certain problems, such as SAs [43]. Third, some data that could impact the management in primary care were unfortunately not measured: SA management by other prehospital providers, information about a management in secondary care,... Finally, results are valid for a non-gatekeeping health system.

## Conclusion

For the first time, we described primary care management at the time of a SA and after the SA in France (a non-gatekeeping health system). GPs were first caregiver following the SA for a minority of patients and they referred to hospitals two-thirds of them. The place of other prehospital providers, such as firemen or family [18] and EDs [15] is more common. Primary care management is important after the SA (more than half patients, more often when GPs were first caregiver following the SA). However, about half patients did not receive care after a SA [22]. Brief contact interventions for any patient leaving the ED after a SA, to help to cope with any new suicidal crisis could be reliable suicide prevention strategies [23, 36]. Connections between GPs and ED upon discharge should be improved.

## Data Availability

At the corresponding authors.

## Abbreviations

GP: General practitioner
SA: Suicide attempt
ED: Emergency department
RR: Relative risk
WHO: World health organization
